# Pharmacogenetics and laboratory medicine: from individual genes towards integral genetic testing

**DOI:** 10.1515/almed-2025-0182

**Published:** 2025-12-05

**Authors:** Mercè Brunet Serra, María Isidoro-García

**Affiliations:** 6567 Section of Pharmacology and Toxicology, Service of Biochemistry and Molecular Genetics, Biomedical Diagnostic Center (CDB), Hospital Clínic, Barcelona, Catalonia, Spain; Institute of Biomedical Research August Pi i Sunyer (IDIBAPS), Barcelona, Spain; Biomedical Research Center in Hepatic and Digestive Diseases (CIBEREHD), Carlos III Health Institute (ISCII), Madrid, Spain; Department of Medicine, University of Barcelona, Barcelona, Spain; Department of Clinical Biochemistry Complejo Asistencial Universitario de Salamanca, Unit of Pharmacogenomic and Precision Medicine, Salamanca, Spain; Institute of Biomedical Research of Salamanca, IBSAL, Salamanca, Spain; Department of Medicine, University of Salamanca, Salamanca, Spain

**Keywords:** pharmacogenetics, laboratory medicine, integral genetic testing

## Introduction

In the era of Personalized Precision Medicine, studies exploring the genetic factors that influence interindividual variability in treatment response, consistently highlighted the need for a comprehensive genetic profile, which led to the development of pharmacogenomics. This holistic approach explores the molecular basis of pharmacological response, leading to the identification of *novel* therapeutic targets. In contrast to pharmacogenomics, pharmacogenetics is focused on exploring the applications of *known* biomarkers of response in specialized clinical laboratories. However, in clinical practice, these two terms are often used interchangeably. In the last decade, multiple research groups involved in the identification and validation of response biomarkers underline the influence of genetics on the pharmacokinetics and pharmacodynamics of multiple drugs [1], [2], [3].

Scientific societies and dedicated consortia for the clinical implementation of pharmacogenomics in national health systems have developed recommendations for personalized prescribing of drug therapies. These recommendations are based on a very high level of evidence of association between pharmacogenes and pharmacological response, both in terms of toxicity and efficacy. In June 2023, the Spanish Ministry of Health presented the Common Catalogue of Genetic and Genomic Tests of the National Health System, which devotes a specific section to pharmacogenomics [4]. Spain was the first country to adopt a measure to ensure equity in access to pharmacogenomics. However, this measure has been implemented unevenly across autonomous communities [5]. A multiplicity of studies provides evidence of the clinical and financial benefits of implementing pharmacogenetic testing [6], 7]. A European multicenter study [8] revealed that preemptive pharmacogenetic testing reduced the occurrence of adverse drug reactions (ADRs) by 20–25 % and contributed to optimizing drug therapies, thereby confirming the clinical benefits of this approach. However, the pharmacogenetic tests currently used to guide clinical decisions are based on a limited panel of actionable gene mutations that explain only 20 % of the variability in observed responses. Other factors, such as drug-to-drug interactions, along with liver and kidney function, age, gender, epigenetics, unknown inheritance patterns, and placebo effect, to name a few, also play a major role in individual response [9]. Big Data emerges as an essential tool for integrating genetic, clinical, and environmental data from vast samples of patients to identify new pharmacogenetic biomarkers. Artificial Intelligence enables the analysis of millions of genetic sequences and other variables associated with pharmacological response. Based on these datasets, Machine Learning is used to generate algorithms for predicting an individual’s response to drugs and to develop support tools for personalized clinical decision-making.

This Editorial provides an overview of the state of the art of pharmacogenetics in clinical practice, its benefits, and the challenges emerging from its implementation, ranging from organizational to digital precision healthcare issues.

## State of art

### Pharmacogenetic approach

Pharmacogenetics can be implemented according to different criteria. When applied to the analysis of specimens, pharmacogenetics is used to test body fluids (such as blood, urine, or saliva) or molecules in tumor tissue, particularly in the context of oncology. Regarding to the type of variant addressed, pharmacogenetics can be *germline*, focused on anticipating patient response (for instance, according to their capacity to biotransform or transport a drug), or *somatic*, focused on assessing tumor response to a drug therapy. According to the number of biomarkers screened for, pharmacogenetics can be categorized into *single-drug* (e.g., specific response to mavacamten) or multidrug (real-world pharmacogenetics) approaches. Considering the point of prescribing, pharmacogenetics is *preemptive* when implemented prior to prescribing a treatment (e.g., DPYD, TPMT genotypes) or reactive when implemented post-treatment. Pharmacogenetics can also be applied in various clinical contexts, such as in the case of transplants, or to assess disease progression in chronic or acute conditions. It is worth noting the role of pharmacogenetics in life-threatening emergencies, such as in Intensive Care, Burn, or Post-Anesthesia Care Units, with the added difficulty of short turnaround times.

### Pharmacogenetics and analytical process

As for all laboratory tests, this Issue addresses the analytical process and emphasizes the need for a Quality Management model, preferably based on the ISO15189 standard, even when the laboratory does not hold ISO certification. With regard to the pre-analytical phase, attention is paid to issues such as the informed consent process, pre-test counseling, and the collection of essential variables, including treatment, epidemiological, and clinical details.

Concerning the analytical phase, focus is placed on the panel of biomarkers to be analyzed according to the criteria aforementioned. The SNS portfolio [4] may facilitate decision-making, as pharmacogenetics should be used to screen for biomarkers supported by a high level of evidence. With respect to analytical methods, emphasis is placed on single-nucleotide variants (SNVs) and copy number variants (CNVs), as in the case of CYP2D6 [10]. Reference is made to the possibility of parallel testing based on mass spectrometry (LC/MS) or next-generation sequencing, or sequential by combining different methodologies, including reverse transcription polymerase chain reaction (RT-PCR), microarrays, or Sanger sequencing. Nevertheless, these processes require the implementation of confirmation systems such as digital PCR.

Regarding the post-analytical phase, the focus is on preparing reports that should be based on guideline recommendations and fulfill all ISO 15189 requirements. Hence, reports should be as comprehensive as possible, including, where available, the patient’s epigenetic and physiopathological details, lifestyle habits, concomitant use of medication, drug-to-drug interactions, and pharmacogenetics. Test results and relevant pharmacogenetic recommendations should be provided by experienced consultants.

### Implementation

The Common Catalogue of Genetic and Genomic Tests of the National Health System establishes a framework that fosters equity and supports the homogenization of healthcare provision. Nonetheless, the implementation of this service has been notably heterogeneous among autonomous communities. The Five-Step Precision Medicine (5SPM) initiative, established in Castilla y Leon, was a successful endeavor involving the integral real-world analysis of treatments administered. This scheme includes digitalization and the implementation of Artificial Intelligence (AI). Other fruitful experiences include the Medea project in Extremadura, which involves population pharmacogenotyping; the Mental Health project of the Xenomic Foundation in Galicia, or the Pharmacogenetics Initiative in the Community of Madrid. Other autonomous communities, such as Valencia and Catalonia, are implementing similar models. An additional example is the Precision Oncology Program offered by the Catalonian Health System.

## Future challenges

Access to training in the fields of analytics, technology, bioinformatics, research, and pharmacology will be essential for the successful implementation of pharmacogenetics. However, these skills are poorly addressed in medical schools, and physicians need to acquire them during fellowships, specialty rotations, or through lifelong learning.

This is a challenging process involving multiple factors, some of which remain unknown. Research is a crucial step in better understanding the molecular basis of diseases and identifying new biomarkers and therapeutic targets. In this context, multiomics will play a major role, including, but not limited to, pharmacoexposomics, focused on understanding the association between exogenous factors and response to medication; pharmacoepigenomics, aimed at identifying the epigenetic factors that influence response to drug treatments; pharmacotranscriptomics to establish the association between RNA expression profiles and treatment response; pharmacoproteomics, aimed at determining the relationship between protein expression and treatment response; or even metabolomics, entailing a comprehensive analysis of the metabolites involved. In a nutshell, the implementation of pharmacogenetics involves the application of Systems Biology to translational research and the knowledge transfer.

Organizational aspects become particularly important, especially in primary care, which provides a comprehensive perspective on therapeutic management and enables the implementation of a real-world approach. In secondary care, special attention should be paid to the living environment of the patient to rapidly screen for specific biomarkers supported by an adequate level of evidence and, where appropriate, adopt a preemptive approach. Additionally, the highly specialized care delivered in the hospital setting should not prevent the adoption of a comprehensive perspective, which highlights the relevant role of multidisciplinary teams.

A major challenge is funding, which is essential for any schedule to be implemented. Human resources are also of paramount importance, as the application of multiomics requires the involvement of highly qualified professionals. In relation to high-throughput technologies, there are ongoing studies investigating next generation sequencing (NGS), previously whole exome sequencing (WES), and currently whole genome sequencing (WGS). Nevertheless, clinical laboratories continue to face considerable challenges, including technical complexity, uncertainty regarding the impact of *de novo* variants, insufficient standardization and validation, and the demanding nature of the certification process.

Specialists should address existing needs to ensure an effective implementation of pharmacogenetics. The transformation of traditional Medicine into Precision Personalized Medicine (PPM) will necessarily involve the application of multiomics, requiring the management of complex facilities, where clinical laboratories will contribute significantly. NGS requires AI-based processing of Big Data and the use of large storage facilities. In addition, pharmacogenetic reports should be prepared by experts who will consider all laboratory data and associated factors that confer it a changing nature; therefore, subsequent re-evaluations and adjustments of recommendations may be necessary over time. This breakthrough in precision laboratory involves the adoption of a multidisciplinary approach where laboratory professionals will play a prominent role in delivering counseling, the driver of transition towards precision medicine.

This revolution in PPM driven by pharmacogenetics has been enabled by significant advances in digital precision medicine, the emergence of Big Data and AI, alongside other innovations including flow management, system interoperability, integration with laboratory information systems (LIS), data interpretation software and, given the multifactorial basis of pharmacological response, the inclusion of telemedicine and wearable-derived data.

Finally, some ethical and legal issues arise from the implementation of pharmacogenetics. Although technology evolves faster than regulations, the summary of product characteristics developed by regulatory agencies facilitates the implementation of pharmacogenetics [11]. Finally, this new approach entails embracing a cultural shift as well as a personal commitment, which healthcare professionals should carefully consider whether they are prepared to undertake.

## Conclusions

Laboratory medicine evolves with advances in PPM. Pharmacogenetics has opened a window of opportunity to improve drug safety and efficacy, providing clear clinical and financial benefits for the National Health System. This transformation process requires standardizing laboratory processes and pharmacogenetic reports. The complexity of all factors influencing interindividual variability in treatment response will require the availability of highly qualified professionals and involve the transformation of clinical laboratories into precision laboratories.
